# Understanding the multifaceted brain mechanisms of acupuncture based on neuroimaging studies: findings and insights from meta-analyses

**DOI:** 10.3389/fnins.2025.1643302

**Published:** 2025-08-19

**Authors:** Beomku Kang, Da-Eun Yoon, Yeonhee Ryu, In-Seon Lee, Younbyoung Chae

**Affiliations:** ^1^Department of Meridian and Acupoints, College of Korean Medicine, Kyung Hee University, Seoul, Republic of Korea; ^2^KM Science Research Division, Korea Institute of Oriental Medicine, Daejeon, Republic of Korea

**Keywords:** acupoints, acupuncture, brain, neuroimaging, mechanism

## Introduction

The interaction between acupuncture treatment and the body is complex and may be mediated by changes in the brain. Acupoint indication and selection demonstrate the connection between disease and acupuncture by showing how acupoints are chosen for specific conditions and the clinical effects of individual acupoints (Hwang et al., 2020a,b). The relationship between disease and brain activity illustrates how acupuncture affects neural activation in diverse pathological conditions. However, due to the variety of brain mechanisms involved in acupuncture treatment, establishing a common brain network to explain its effects has been challenging (Kang et al., 2025b). The relationship between acupuncture and brain activity demonstrates how different acupoints influence neural responses. Investigation of brain patterns induced by stimulation of specific acupoints has tried to reveal acupoint-specificity in acupuncture stimulation across different conditions (Huang et al., 2012; Chae et al., 2013). Therefore, understanding the role of brain imaging is crucial for elucidating how acupuncture affects the body and brain.

Over the past two decades, research has investigated the relationship between acupoints and brain activity using neuroimaging techniques (Zhang et al., 2020; Wang et al., 2025). The first neuroimaging study on acupuncture reported that specific acupoints might induce corresponding responses in the brain, implying relationships based on acupoint characteristics. They found that activation of the brain cortex correlated with acupoint stimulation. However, this article was retracted by the authors, who reported that no acupoint specificity exists for pain and analgesia. Kong et al. rejected acupoint specificity, noting that vision-related acupoints (BL60 and GB37) did not induce specific brain signals in the occipital cortex (Kong et al., 2009). A meta-analysis of neuroimaging on acupuncture found that brain activity reflects pain processing from needle insertion (Chae et al., 2013). Overall, there is no consensus on acupoint specificity in brain imaging. Nevertheless, numerous studies have investigated the brain activation patterns in response to specific acupoints and have explained the mechanisms underlying their action.

This article summarizes three meta-analyses that examined activation patterns after stimulating three major acupoints: LI4, LR3, and ST36. We address concerns interpreting the activation patterns and propose directions for future neuroimaging research.

## Brain-activation patterns associated with major acupoints

Gao et al. conducted a meta-analysis of brain activity following needling at LI4; they observed activation of the postcentral gyrus and deactivation of the anterior cingulate cortex and superior temporal gyrus (Gao et al., 2022). The activation of the postcentral gyrus was linked to the sensorimotor network, while the deactivation of the anterior cingulate cortex and superior temporal gyrus was associated with the limbic system. However, the limbic-paralimbic-neocortical system, a network involved in emotion and cognition, exhibits similar activation patterns in response to stimulation at other acupoints, such as LR2, LR3, ST44, and ST36 (Fang et al., 2009). Thus, acupuncture may target a general mechanism of action, specifically the limbic-paralimbic-neocortical system, as reflected in functional brain imaging.

Huang et al. and Zhang et al. reported distinct brain-activation patterns following ST36 stimulation (Huang et al., 2022; Zhang et al., 2023). They observed activation in the opercular part of the right inferior frontal gyrus, left superior temporal gyrus, right median cingulate gyrus, left and right cerebellum, bilateral Rolandic operculum, and right supramarginal gyrus. At the network level, activation was noted in the auditory, anterior, and posterior salience networks. These findings demonstrate that acupuncture stimulation activates areas in the somatosensory and cognitive regions as well as areas associated with visceral pain. These findings demonstrate specific brain-activation patterns for different acupoints.

**Figure 1:**
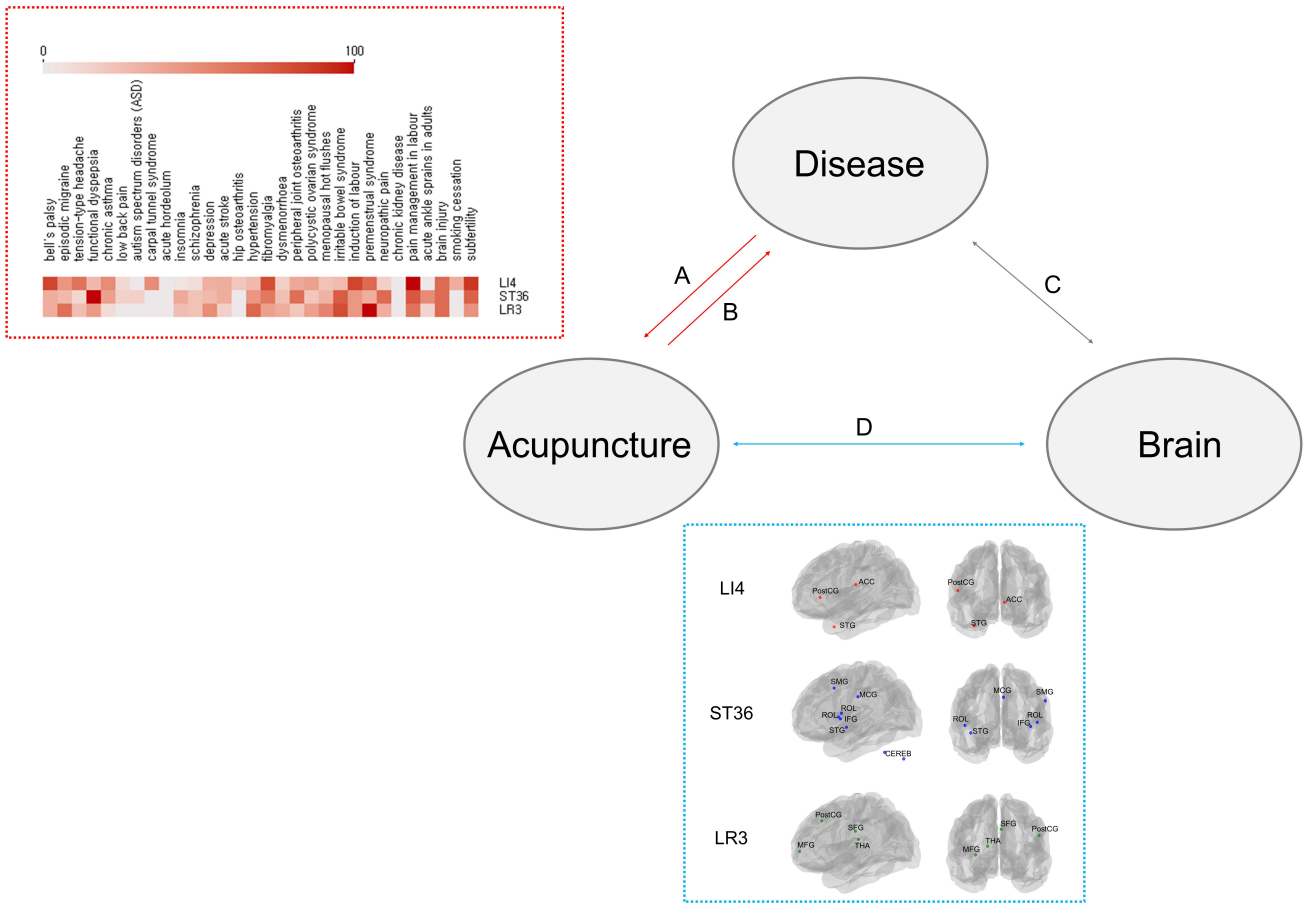
Brain-activation patterns and acupoint indications. Lines A and B represent how acupoints are selected when treating specific diseases, which can help clarify how a given point may function across various pathological conditions. Line A: acupoint selection (forward inference), Line B: acupoint indication (reverse inference). Line C represents the relationship between disease and neural responses. Line D represents the relationship between acupuncture and neural responses. Red box shows acupoint indication patterns for LI4, ST36, and LR3. Data on frequently associated diseases were extracted from the Acusynth database, which catalogs acupoint selection patterns across various conditions. Blue box shows brain-activation maps for acupoints LI4, ST36, and LR3. These maps were generated using data from published meta-analyses, and visualized with the Nilearn Python library (https://nilearn.github.io/stable/index.html). ACC, anterior cingulate cortex; PostCG, post-central gyrus; STG, superior temporal gyrus; SMG, supramarginal gyrus; MCG, median cingulate/paracingulate gyrus; ROL, Rolandic operculum; IFG, inferior frontal gyrus; CEREB, cerebellum; MFG, middle frontal gyrus; SFG, superior frontal gyrus; THA, thalamus.

Rao et al. reported activation in the right postcentral gyrus, left thalamus, left middle frontal gyrus, and right superior frontal gyrus following acupuncture stimulation at LR3 (Rao et al., 2024). They also found that the duration of acupuncture was positively correlated with the activation of certain brain areas and that the *de-qi* sensation (a feeling of numbness, tingling, or heaviness) was more crucial for clear activation of brain regions than needling depth or needle diameter. These results indicate that acupuncture operates through general mechanisms, such as the primary somatosensory cortex or the lateral/medial thalamocortical pathway. The brain-activation areas are shown based on MNI coordinates (Figure 1, see blue box).

## Interpretation of brain-activation patterns associated with major acupoints

We compared three acupoints (i.e., LI4, ST36, and LR3) using data from Acusynth, a database that compiles acupoint selection patterns for various diseases (Lee et al., 2022b). We found that LI4 is widely used across most conditions, and is primarily associated with pain relief. ST36 is also frequently utilized across various conditions, reflecting its role in pain relief and mood regulation. By contrast, LR3 has lower usage overall, mainly associated with premenstrual syndrome and pain disorders (Figure 1, see red box). These acupoints are recommended for pain control to maximize general effects (Lee and Chae, 2021b).

However, the question still remains whether a specific brain response indicates a mechanism intrinsic to an acupoint? To address this, we need to consider the concepts of forward and reverse inference. Forward inference is the act of inferring function from brain region activation, and reverse inference is the act of inferring cognitive process from such activation (Poldrack, 2006).

For example, LI4 is reported to cause deactivation of the anterior cingulate cortex and the superior temporal gyrus. Through forward inference, we can hypothesize that LI4 is associated not only with the deactivation of the limbic-paralimbic-neocortical system (a network involved in emotion and cognition) but also with the activation of other regions, such as the postcentral gyrus. In reverse inference, activation of this system is observed with various acupoints, such as LR2, LR3, ST44, and ST36 (Fang et al., 2009). Therefore, we can infer that specifically activated brain regions may be related to multiple acupoints.

Many of the studies that have reported brain activation following acupoint stimulation referred to neural mechanisms that have previously been identified. For example, stimulation of LR3 has been shown to activate various regions associated with pain processing. However, this does not imply that the neural mechanism of LR3 is limited to pain processing alone; there may be additional pathways that it stimulates. Similarly, other acupoints can elicit brain responses through the pain-processing pathway. These findings imply that neuroimaging data resulting from acupoint stimulation do not represent a complete neural mechanism, but rather a component of a broader, yet uncertain mechanism.

## Considerations for future research

To comprehend how neural responses can reveal insights into the mechanisms underlying the effects of acupuncture, we should first consider what acupuncture signifies to our body. Acupuncture consists of two components: the stimulation itself and the location of this stimulation.

Understanding acupuncture as a form of sensory stimulation is crucial. The procedure acts as somatic input, afferent stimulation received through mechanoreceptors that travels through the spinal cord and brainstem and is relayed from the thalamus to the somatosensory cortex (Chae et al., 2013; Napadow et al., 2019). However, it is subject to various forms of cognitive modulation such as placebo effects or rewarding effects (Lee and Chae, 2021a). In addition, recipients may perceive it as nociceptive input when it was not in clinical context (Lee et al., 2015).

Regarding the location of stimulation, these are the acupoints, but their nature remains a controversial issue (Kang and Chae, 2025). They serve an important role in identifying and naming anatomical areas used by acupuncturists, but whether they have significance beyond that is still debated (Lee et al., 2022a). Based on current evidence, there do not appear to be specific mechanisms unique to each acupoint. Instead, acupuncture seems to function more generally. As mentioned earlier, acupuncture is a form of tactile stimulation and is influenced by cognitive modulation, target disease, and other factors. Thus, brain responses to acupuncture are a complex interplay of various factors intrinsic to acupuncture (Kang et al., 2025a). While analysis of neuroimaging data may provide insights into how acupuncture affects the body and brain, interpreting these results remains challenging.

## Conclusion

This report highlights concerns regarding the interpretation of acupuncture-induced activation patterns in the brain revealed by neuroimaging. We also propose future research directions. Neural responses to acupuncture are influenced by various factors, including cognitive modulation and the disease target, implying that while neuroimaging data can provide valuable insights, interpretation requires caution and should not be considered definitive evidence of the mechanisms underlying acupuncture.
